# An Introduction to *Diopatra*, the Amazing Ecosystem Engineering Polychaete

**DOI:** 10.3390/biology12071027

**Published:** 2023-07-21

**Authors:** Andrés Arias, Sarah A. Woodin, Hannelore Paxton

**Affiliations:** 1 Department of Organisms and Systems Biology (Zoology), University of Oviedo, 33071 Oviedo, Spain; 2 Department of Biological Sciences, University of South Carolina, Columbia, SC 29208, USA; woodin@biol.sc.edu; 3 School of Natural Sciences, Macquarie University, Sydney, NSW 2109, Australia; hannelore.paxton@mq.edu.au; 4 Australian Museum Research Institute, 1 William Street, Sydney, NSW 2010, Australia

**Keywords:** Annelida, Onuphidae, morphology, reproduction, ecology, fishing bait

## Abstract

**Simple Summary:**

The genus *Diopatra* is a major driver in sedimentary systems, altering the structure of habitats and changing the frequency of refugia and predator access to prey. It is taken as prey by a variety of shorebirds, crustaceans, and fish and used worldwide as bait. *Diopatra* are quite charismatic, with iridescent colour patterns and a willingness to be fed by hand by entranced biologists, and their larvae can only be described as ‘cute’. One might expect then that given their importance to sedimentary systems, the bait trade, etc. that we would know more than we do about their reproductive modes, physiological tolerances, etc. than we do. The recent discovery that the predominant onuphid of the very well-known Atlantic coast of Europe, *D. neapolitana*, is a protandric sequential hermaphrodite is startling. This special volume dedicated to *Diopatra* will hopefully stimulate more investigations and further insights.

**Abstract:**

The annelid genus *Diopatra* occurs in all major oceans but is best represented in the shallow depths of warmer waters, where it lives in elaborately decorated tubes. This paper provides an introduction to the animals, discussing their history and diversity. We describe and illustrate its morphology and geographic distribution. While they were thought to be predominantly gonochoristic, recent reproductive studies show that several species are protandric simultaneous hermaphrodites. Development is by broadcast spawning with a brief pelagic stage or direct development in the parental tube or egg mass attached to it. *Diopatra* is a key ecosystem engineer, altering water flow and deposition and increasing the availability of refugia. We also discuss its harvesting as fishing bait, its role as an alien or introduced species, its capacity to regenerate, its therapeutic potential, and its applications as a bioindicator species for climate change, geographic distribution changes, and dispersal.

## 1. Introduction

Bristle-worms or polychaetes are marine annelids that occur from the littoral zone to the deepest trenches, inhabiting soft and rocky bottoms or leading a pelagic life. Among them, members of the family Onuphidae Kinberg, 1865 [1] are among the most important polychaetous annelids in soft sediment communities worldwide. Members of the genus *Diopatra* Audouin and Milne Edwards, 1833 [2] have been coveted as fishing bait for almost two centuries and are known as ecosystem engineers, stabilizing sediments with their tubes and therefore increasing the structural complexity and biodiversity of their infaunal habitat [3]. *Diopatra* has attracted attention in the past decade not only for its surprising and unknown diversity [4,5,6], but also for its role as an indicator of climate change [7,8], a sentinel group for drugs in marine environments [9], and an example of protandric hermaphroditism [10,11,12].

The aim of the present paper is to provide an overview of *Diopatra* and serve as an introduction to this volume.

## 2. History and Diversity

The first named species was *Nereis cuprea* Bosc, 1802 [13] from South Carolina, USA, stated to be very common in the area of Charleston. It was grouped with the ‘‘Néréides à bouches armée de mâchoires’‘ (nereids armed with a toothed mouth), and in 1833, it was transferred to the newly erected genus *Diopatra*. The presence of branchial filaments, arranged in a spiral around the central branchial trunk, is the main defining character of *Diopatra*, one of the oldest and most beautiful genera of polychaetes of the family Onuphidae. It is one of the few polychaete genera that is defined by a single characteristic and the status of which has never been amended or changed. 

It is the largest known genus of the family, represented by 67 recognized species worldwide, of which 21 were described before 1900, 43 between 1900 and 2000, and 13 in the new millennium [14]. Although the genus is uniquely defined by its autapomorphy of possessing spiralled branchiae, specific identification is notoriously difficult as they are superficially very similar and lack clear diagnostic features. They can be distinguished only by combinations of characteristics that show various degrees of overlap and variability. This problem of species delineation has been recognized for a long time [15,16,17,18] and even led the famous French polychaetologist Pierre Fauvel [19] to make the statement that most described species of *Diopatra* constitute a single variable worldwide species, namely *D. neapolitana* (Delle Chiaje, 1841) [20].

Notable taxonomic revisions [16,17,21,22,23] have led to the recognition of new diagnostic characteristics, to which the advent of scanning electron microscopy (SEM) has contributed greatly. Most recently, new morphological character sets have been explored and evaluated including parapodial lobes and folds and maxillary characteristics [6]. However, the most significant aid to phylogenetic analyses came with integrative studies and the application of sequence-based genetic methods. The earliest genetic analyses investigated the identity of Western European species [4,7,12,18] and South American faunas [6]. The present SI will expand it to West African and North and South American *Diopatra* diversity (see papers by Hektoen et al., 2021; Sotka et al., 2023) [24,25].

## 3. Morphology

**Figure 1:**
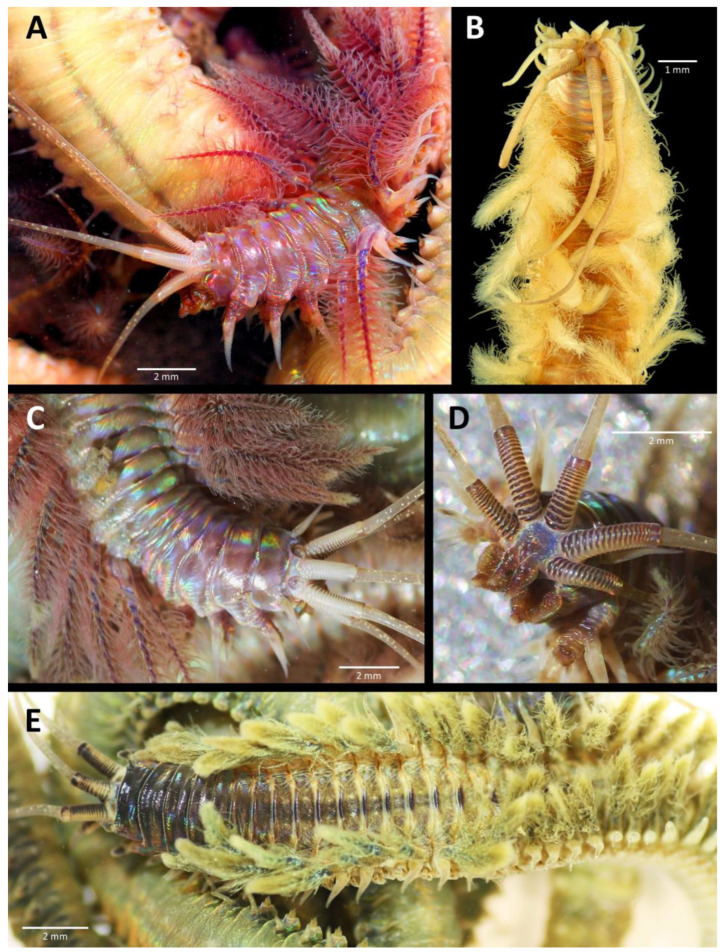
Photographs of *Diopatra* spp. living specimens (anterior ends): (**A**) *D. aciculata*, lateral view; (**B**) *D. gallardoi*, dorsal view; (**C**) *D. aciculata*, dorsal view; (**D**) detailed view of the prostomium of the same; (**E**) *D. neapolitana*, dorsal view.

*Diopatra*, with its often bright colouration and spiralled branchiae, is one of the most beautiful onuphid genera (Figure 1). The smallest species measure only a few centimetres in length while a large live *D. neapolitana* can be 80 cm long with almost 400 chaetigers and over 1 cm in width [12]. The anterior part of the body bears a small head or prostomium with highly developed sensory structures (Figure 1D), followed by a strongly muscularized anterior region, grading into a softer median and posterior end. The animals are adapted to a tubicolous existence in which the anterior body can be partly everted from the tube for feeding and tube construction but can be rapidly withdrawn into the protective tube at the slightest signs of danger.

**Figure 2:**
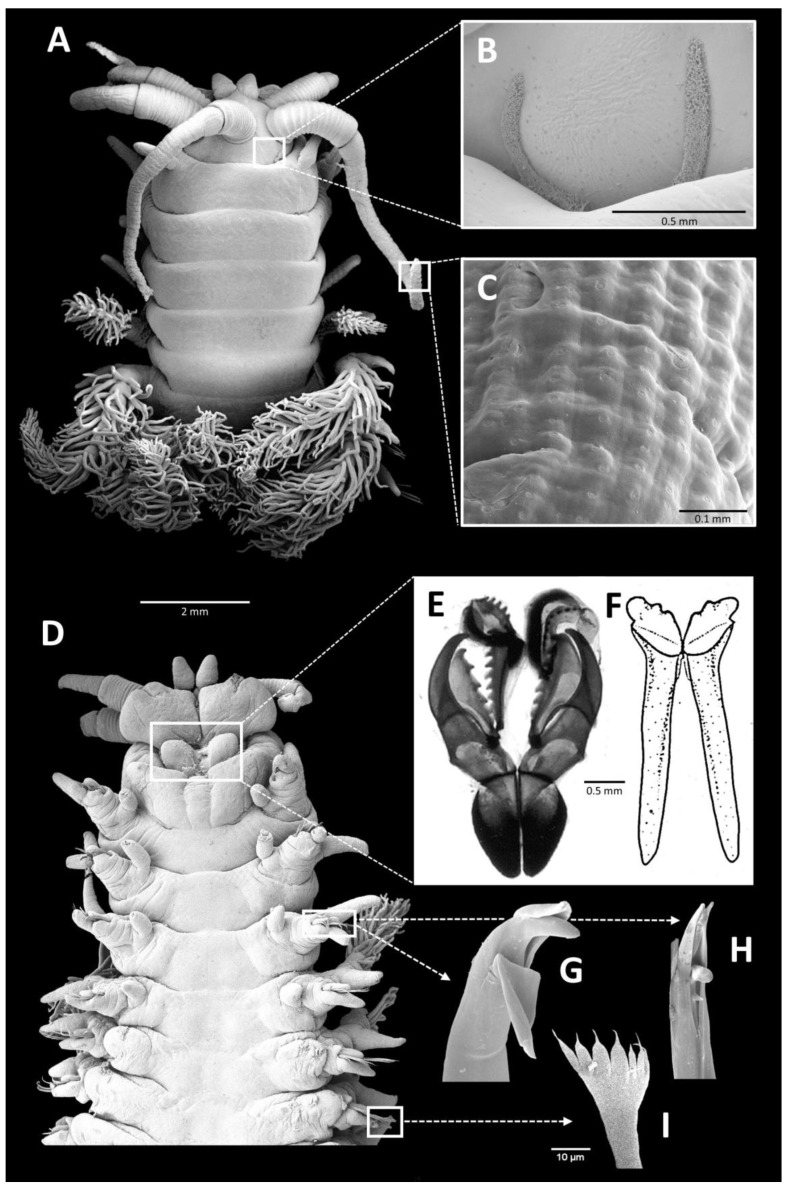
*Diopatra neapolitana*, scanning electron micrographs/drawings: (**A**) anterior end, dorsal view; (**B**) detailed view of nuchal groove; (**C**) detailed view of the sensory buds of antennae; (**D**) anterior end, ventral view; (**E**) maxillae; (**F**) mandibles; (**G**) falcate or unidentate moderately robust simple hook from modified parapodium; (**H**) bidentate lower slender pseudocompound hook from same; (**I**) pectinate chaeta.

The prostomium (Figure 2A) bears a pair of anterior sensory lips and five appendages with basal ringed ceratophores and distal ceratostyles. The two anterolateral appendages are palps, and the inner three are antennae. The ceratostyles are covered in combined sensory/secretory structures termed ‘Sinnesknospen’ or sensory buds [26]. Pflugfelder described their histology, showing that cilia project through the cuticle from a central sensory cell and that the ciliated cell is accompanied by one or two serous glands opening to the surface [26]. Although present in all onuphids, these are arranged in conspicuous rows in *Diopatra* (Figure 2C). Small eyespots are only present in juveniles; oval to almost circular structures that had previously been interpreted as eyes, located at the posterior part of the prostomium, are nuchal organs forming ciliated grooves (Figure 2B).

The anterior chaetigers (Figure 2) are modified for quick propulsion from and retraction back into the tube. The well-developed longitudinal muscles provide the power, aided by the modified enlarged parapodia, which are directed anterolaterally and bear specialized hooks. The branchiae commence from parapodia 4 to 5 (Figure 2A,D) and reach their greatest development by about chaetiger 20. Thereafter, the filaments become reduced and are absent at about chaetiger 50–70. The median and posterior part of the body is basically a container for gut and reproductive products.

As an onuphid, *Diopatra* belongs to the order Eunicida, possessing a complex jaw apparatus consisting of a pair of ventral mandibles and dorsal maxillae (Figure 2E,F). The jaws are hardened cuticular structures, composed of calcium carbonate and/or scleroproteins. As a result of their durability, they have a good fossil record. While the earliest known eunicidan jaw elements of extinct families date from the latest Cambrian, fossils of the extant Onuphidae have not been identified but would be expected to be much younger [27,28]. Onuphid jaws become visible in three-day-old larvae. While the larval mandibles are retained and added on throughout the lifetime, the maxillae change to a brief juvenile version before the adult stage that will thereafter moult periodically to achieve growth [29]. The jaw kinematics of *Diopatra* spp. have been observed by filming individuals biting, and a comparison with *Lumbrineris* spp. was interpreted to be consistent with their differences in diet [30].

## 4. Geographical Distribution

The genus occurs in all major oceans but is best represented in warmer waters, where it is found in shallow depths. However, two specimens of *Diopatra* sp. from South Georgia Island, Antarctica have been reported from a depth of 217 m [31]. Although more than 60 species are recognized, many are only known from their original descriptions. Some of the best-known names, such as *D. neapolitana*, *D. amboinensis* Audouin and Milne Edwards, 1833 [2] and *D. cuprea*, are credited with worldwide distributions that are not trustworthy [25]. 

**Figure 3:**
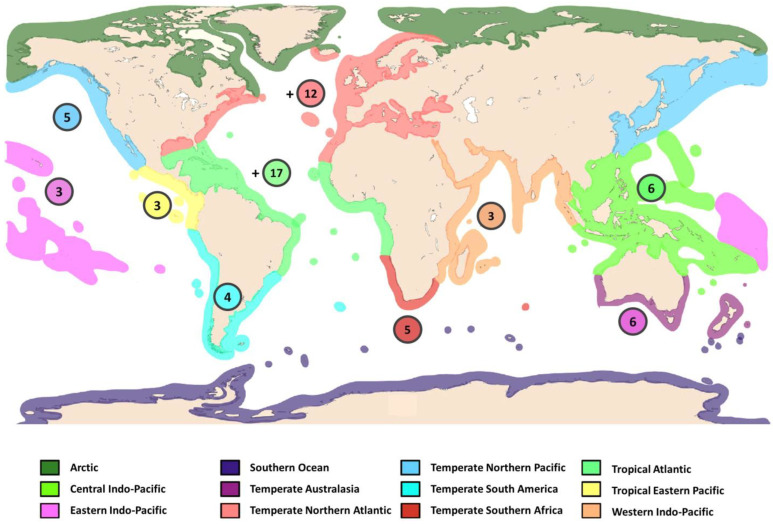
Distribution of the recognised *Diopatra* spp. by bio-geographical region (Marine Ecoregions of the World). + indicates evidence of the existence of undescribed species from the region. Data from WoRMS. Map adapted from Smit et al. [32].

Figure 3 presents an overview of the distribution of the type localities of the 65 recognised *Diopatra* species and their biogeographical regions. Intense interest and activity in regional faunal studies commenced during the last decades. The most remarkable knowledge increase in the diversity and reproductive biology of *Diopatra* is from European waters. Whilst *D. neapolitana* was thought to be the only accepted European representative of the genus until quite recently [8,18], they presently number ten, with seven species from France, Portugal, and Spain described since 2010. The most diverse region is Macaronesia, a region comprising five eastern North Atlantic archipelagos: the Azores, Madeira, the Salvage Islands, the Canary Islands, and the Cape Verde Islands from which nine of the ten species have been reported [5]. 

West Africa is a very rich area that has been previously studied [15,33,34] and where new active studies are in progress. An integrated unpublished master’s study discovered 14 species of *Diopatra*, of which five were previously known and nine were new to science. Some of this work has been presented as a paper in this SI (see Hektoen et al., 2021) [24].

North America has a surprisingly small representation of *Diopatra* species, with only *D. cuprea* reported from its eastern coast [35]. However, this is an underrepresentation, since the famous *D. cuprea* represents a species complex consisting at least of five lineages (see paper by Sotka et al., 2023 in this SI) [25]. Nine species have been reported from the western USA and Mexico, largely as a result of the studies by Olga Hartman and Kristian Fauchald [16,21]. 

In the southern hemisphere, *Diopatra* fauna are well represented in Australia, where nine species have been reported [17]. Another centre of a rich *Diopatra* history is Brazil, where the reported 14 species from the first half of the 20th century were established by European workers. However, only half of these are presently accepted, but a keen group of polychaetologists is studying these fauna with integrated methods, which has recently resulted in the description of four new species and the strong suggestion that the range of *D. cuprea* may extend southward only into the Caribbean Sea [6]. 

## 5. Reproduction and Development

*Diopatra* species are annual breeders with discrete or interrupted breeding seasons. They were thought to be predominantly gonochoristic, as ripe specimens could be distinguished by the colour of their sexual products through their body wall; eggs were yellowish to greenish and sperm whitish to cream-coloured. However, an intensive study of *D. neapolitana*, combining field observations with a histological study of monthly collected individuals, revealed that the species is a sequential hermaphrodite of the protandry type. The studied Spanish population consisted of pure males in the smallest-sized class, hermaphrodites in the medium-sized classes, and pure females in the largest ones [12]. External detection of sperm or eggs only confirms the dominant sexuality of individuals at one time point. The reproductive cycle of *D. neapolitana* in northern Spain and its timing of development is fully described in Arias et al., 2016 [12]. See also the study by Escobar-Ortega et al. [36] in this SI, suggesting that water temperature is one of the most important drivers of the reproductive cycle for this species. 

Further studies on other species of *Diopatra* suggest that the hermaphroditic condition may be widespread in this genus [10,11]. Another European *Diopatra* species, *D. biscayensis* (Fauchald et al. (2012)) [37] from northern Spain and France is a protandric simultaneous hermaphrodite [11]. This has also been suggested by Arias et al. (2013) [10] for the presumed gonochoristic population of *D. marocensis* (Paxton et al., 1995 [38]) from the Portuguese coasts studied by Pires et al. (2012) [39]. The sequential hermaphroditism can be explained by the ‘size advantage’ hypothesis, originally developed by Charnov (1982) [40] and later expanded by Ghiselin (1987) [41]. This hypothesis postulates that the reproductive success of an individual as a male or as a female is closely linked to its body size or age and that the relationship between reproductive success and size/age differs for each sex. Thus, protandry is expected when a large body size increases female fecundity more than male fertility. Protandry is the most common type of sequential hermaphroditism among polychaetes and marine invertebrates [42]. In *Diopatra*, a larger female can produce more eggs than a small female, and since they are broadcast spawners with random fertilization, the reproductive success of small and large males is likely to be almost the same, and this suppresses the advantages of protogyny. 

Other studies of the reproductive biology of *Diopatra* spp. (mainly of *D. neapolitana* from different locations, e.g., [43,44]), despite assuming the gonochoristic condition of the species, have concluded unambiguously that within the studied populations, the smallest female found was larger than the smallest male and the male:female ratio deviated from the expected Fisher 1:1 sex ratio for gonochoristic or dioecious species. These observations strongly suggest protandry, leading us to consider the possibility that sequential hermaphroditism is largely underreported for *Diopatra* species. Other studies in a smaller species of *Diopatra*, *D. marocensis*, revealed that this species was hermaphroditic. However, within the studied northern Iberian populations, no pure males or females were found, and the species was considered a simultaneous hermaphrodite [10]. This type of hermaphroditism was previously reported in *Diopatra* sp. from Sumatra [45]. Simultaneous hermaphroditism is typically correlated with brooding behaviour, direct or lecithotrophic development, and a sedentary habit [10,41]. Although these relationships are not very well defined, many members of the genus *Diopatra* satisfy these requirements [17]. Consequently, histological reproductive studies should be extended to more species within the genus which remain poorly known biologically.

**Figure 4:**
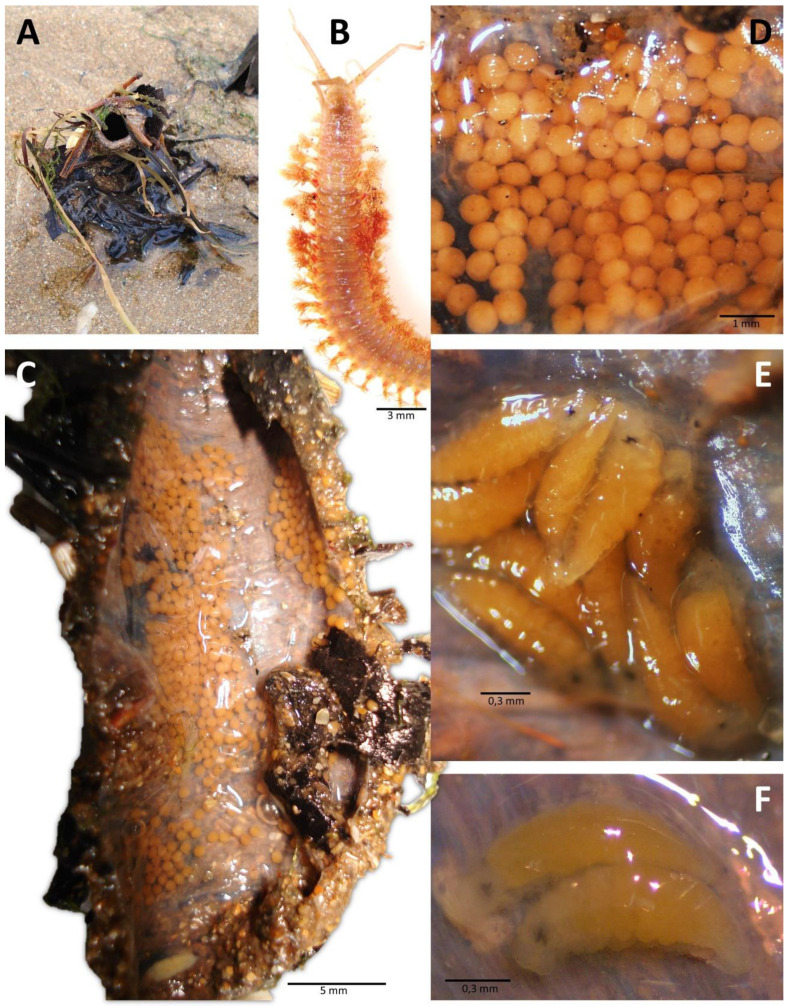
Early development of the brooder *D. marocensis* from Villaviciosa estuary, Bay of Biscay: (**A**) protruding portion of tube in nature; (**B**) anterior end of mature worm; (**C**) tube dissection showing the brood; (**D**) detailed view of brooded fertilised eggs; (**E**) brooded juveniles of 8–10 chaetigers; (**F**) brooded juveniles of 12 chaetigers.

Paxton (2016) [17] summarized the known patterns of larval release or retention across the genus. She described four known patterns, including (1) brooding within the tube, typical of small species presumably for protection of gametes and juveniles; (2) deposition of eggs not inside the parental tube but in a gelatinous matrix or sac attached to the distal end of the tube where initial development occurs; and (3) direct release without a retention stage. The latter two are typical of larger species such as *D. neapolitana* and *D. biscayensis* [11,12]. Table 1 is an updated version of Paxton’s original table. Two observations are immediately obvious. First, brooding may be to a very late stage juvenile such as those of *D. marocensis* Paxton et al., 1995 [38] (Figure 4) and *D. tuberculantennata* Budaeva and Fauchald, 2008 [46] which were collected with 34- and 28-chaetiger juveniles in adult tubes respectively [46,47]. Juvenile *D. marocensis* with 32–40 chaetigers were collected with adults in their own tubes in the sediment, indicating that once they leave the parental tube, they do not disperse any further but settle and build their own tubes among the adults [38]. Our knowledge of the worms’ development in the tube is very limited as in most cases, the young collected in the tube are very small and consist of 6- to 15-chaetigers (Figure 4E). These are collection snapshots and, like those of adults with reproductive products, they do not reveal the full temporal pattern. 

**Figure 5:**
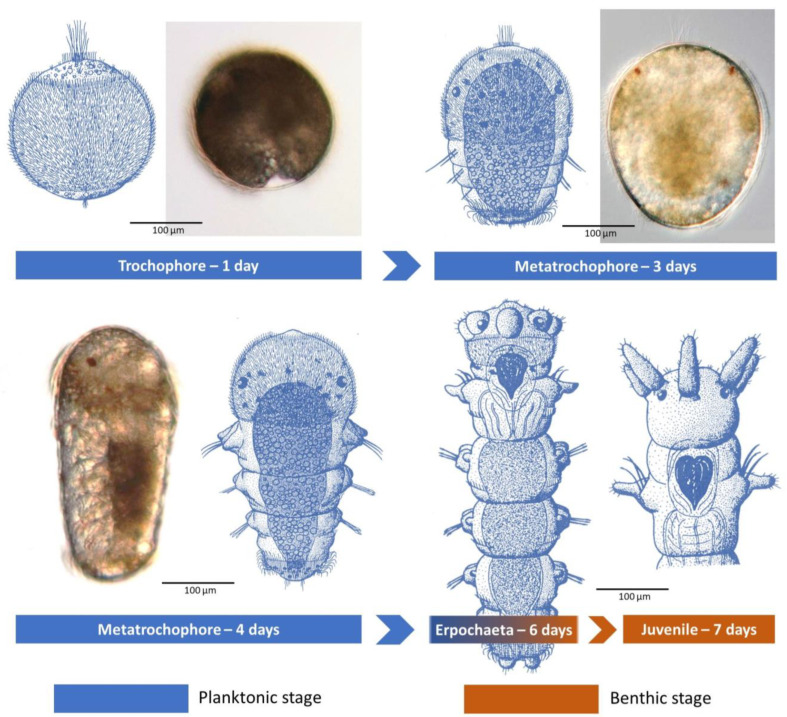
Larval development up to juvenile stage of *D. neapolitana* from the Bay of Biscay. Line drawings modified from Cazaux [49] and micrographs modified from Bergamo [50].

Second, it would appear that the planktonic period is quite short, two to four days, or non-existent for those with either a short duration egg mass external to the tube lumen or perhaps direct broadcast spawning (Table 1). Laboratory studies of *D. cuprea* by Allen (1959) [48] and of *D. neapolitana* by Cazaux (1970) [49] are still the classical references, with their detailed descriptions and illustrations of short-lived, free-swimming lecithotrophic larvae (Figure 5). In each case, the larvae have small eyespots and consist of 3–6 chaetigers by day three to five, when they start to settle and build their own tubes (Table 1; Figure 5).

**Table 1:** Presence of a planktonic period in species of *Diopatra*. ~67 known species of *Diopatra*; only 14 with known larval type, i.e., brooded or released. Species marked with an asterisk are known to be hermaphroditic. Egg size in µm.

Species	Locale	Egg Size	Planktonic Period	Reference
*D. aciculata*	S Australia	230	4–5 days (settle at 4–6 chaetigers)	Paxton and Safarik [29]
*D. albimandibulata*	Queensland, AU	300	brooding 3-chaetiger larvae	Paxton [17]
*D. biscayensis* *	Bay of Biscay, SP	260	none to short, 1–2 days (released at 4–5 chaetigers, some +phototactic)	Arias and Paxton [11]
*D. cuprea*	NE USA	240	3–4 days (settle at 4 chaetigers)	Allen [48]
*D. gigova*	W Australia	1400	none, brooding	Paxton [17]
*D. lilliputiana*	W Australia	400–700	none, brooded to ≥15 chaetigers	Paxton [17]
*D. maculata*	W Australia	350	gelatinous egg mass on outside of tube with 3–4-chaetiger larvae	Paxton [17]
*D. marocensis* *	Morocco, Aveiro PT	600-620	none, brooded to ≥23–34 chaetigers	Fadlaoui et al. [47], Pires et al. [39]; Arias et al. [10]
*D. neapolitana* *	Arcachon FR, Sardinia IT, Aveiro PT, N Spain, NW Spain	240	3–4 days (settle at 3–5 chaetigers)	Cazaux [49], Conti and Massa [51], Pires et al. [39], Arias et al. [12], Escobar-Ortega et al. [36]
*D. nishii*	Japan	600–700	none, brooded to ≥21 chaetigers	Paxton [17]
*D. ornata*	Catalina Island CA, USA	235	4 days	Emerson [52], Fauchald [53]
*D. sugokai*	Maeshiba, Japan	200	3 days (settle at 5 chaetigers)	Choe [54]; Paxton [17]
*D. tuberculantennata*	Belize	?	none, brooded to ≥25 chaetigers	Budaeva and Fauchald [46]
*D. variabilis*	Madras, India	600	none, brooded to ≥15 chaetigers	Krishnan [55]

## 6. Ecological Roles

**Figure 6:**
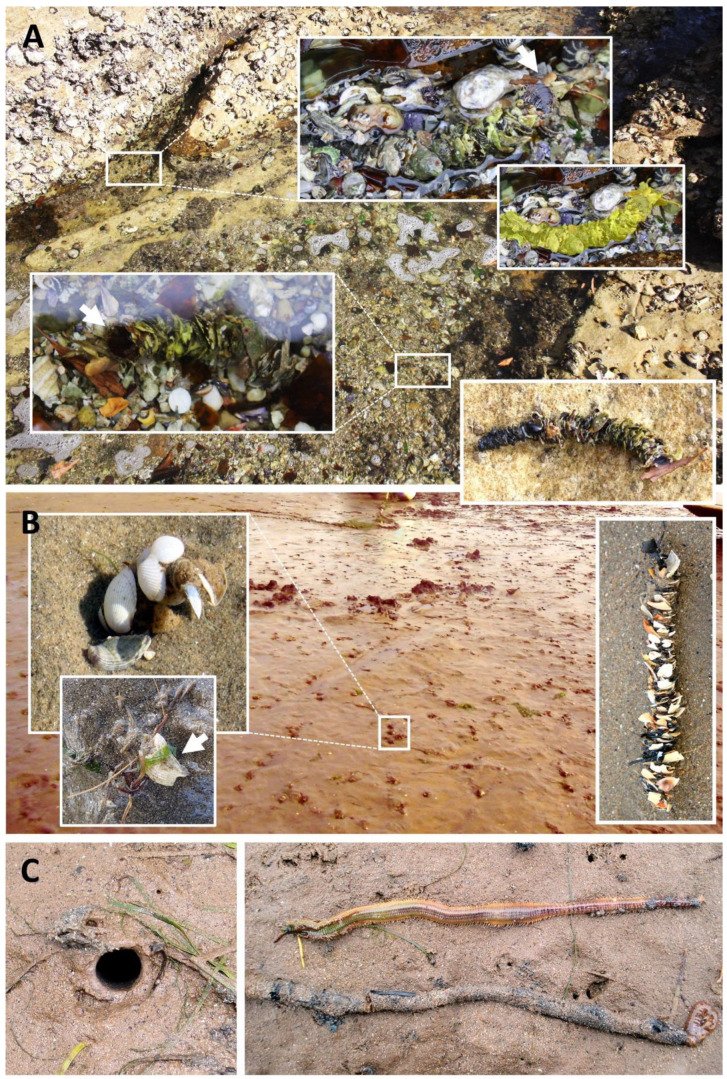
*Diopatra* spp. habitats and tube types: (**A**) *D. dentata* from the rocky shore near Bondi (Sydney, SE Australia); (**B**) *D. biscayensis* from the sandy coves of Nouvelle-Aquitaine (Atlantic France, Bay of Biscay); (**C**) *D. neapolitana* from the sandy flats of Villaviciosa estuary (northern Spain, Bay of Biscay).

The worms are tubicolous, building vertical tubes in the sediment that may extend their upper portion (known as the tube-cap) a few centimetres above the sediment (Figure 6). Their tubes vary from having scarcely any ornamentation, being mostly composed of silt and fine sand (Figure 6C), to being exquisitely ornamented with shells, seaweed, or any foreign material (Figure 6A,B), and often serve as good field characters as to species [11,51,52,53,54,55,56]. Almost unique to *Diopatra* is that materials such as shells are attached edge-on, imbricately, rather than flat, as in most tube-builder constructions.

Mangum and Cox (1971) [57] investigated the feeding modalities and responses to chemical stimuli of *D. cuprea*, which has an extensively decorated and emergent tube-cap; its diet had been investigated previously by Mangum et al. (1968) [58], who reported seeing individuals browsing on material on the tube-cap and thought the tube-cap served in part as a food-catching device. Similarly, Brenchley and Tidball (1980) [59] showed that individuals of *D. cuprea* orient their tube-caps perpendicular to unidirectional flows, but under conditions with high densities, tube-caps are oriented parallel or perpendicular to one another and *D. cuprea* browse on one another’s tube-cap community. Shells being attached imbricately is known to increase the ability of *D. ornata* Moore, 1911 [60] to avoid predatory attacks, while attached algae do not [61]. The shell also changes the composition of the tube-cap community of *D. cuprea* [62]. Interestingly, *Diopatra* do not appear to discriminate between shell and algae as attachment material [61], and contrary to popular belief, tube-cap decoration does not appear to impart crypsis [63].

*Diopatra cuprea* and other *Diopatra* with emergent tube-caps act as ecosystem engineers by stabilizing the sediment with their tubes and altering flow dynamics with emergent tube-caps, therefore increasing the structural complexity and biodiversity of their infaunal habitat [3,64]. Additionally, the tubes and emergent tube-caps of *Diopatra* when dense provide shelter from disturbance and predation [65] and may actively facilitate the attachment of some seaweed species or other fouling fauna that cannot survive on soft bottoms without these structures [66,67,68]. Furthermore, *Diopatra* constitutes an important food source for many species, such as crabs, fish, and birds, as well as acting as a host for parasites [69,70,71,72].

Although the worms can be solitary, they often occur in aggregations that have been reported to be up to 21,800 per m^2^ of *D. dexiognatha* Paxton and Bailey-Brock, 1986 [73], a 1.5–5 cm worm, on the shore of Oahu, Hawaii [64,73]. At densities over 6 per 0.01 m^2^ of *D. cuprea*, a worm 10 to 20 times larger than *D. dexiognatha*, predation, disturbance, and erosion are dramatically altered [3,65], leading to increased infaunal abundances. Shorebirds, large crabs, *Limulus*, and some fish are all inhibited by such densities of *Diopatra*, while rays are not (Luckenbach, 1984; Woodin et al., 2019) [74,75]. A review of the ecology of *Diopatra* is presented in this SI (see paper by Berke, 2022) [76].

## 7. *Diopatra* spp. as Fishing Bait

Already in the original description of one of the first species of the genus, *D. neapolitana* (Figure 1E and Figure 5C), a mention of its value as fish bait is highlighted [20]. From the initial harvest of large species such as *D. neapolitana* and *D. aciculata* Knox and Cameron 1971 [77] (Figure 1A,C,D) by local fishermen, this activity has grown into a considerable industry, with live bait being shipped throughout Europe, Asia, and Australia [11,12,78,79]. In southwestern Europe (mainly Spain, France, and Portugal), the species most commonly harvested and subsequently sold for fishing and surfcasting are *D. neapolitana* and *D. biscayensis* (Figure 6B,C), sometimes mixed with *D. marocensis* (Figure 6C), since in some Bay of Biscay estuaries the three species occur sympatrically [11]. Along the coast of the Mediterranean, *D. neapolitana* is harvested intensively [44]. In Turkey and Portugal, field populations appear seriously impacted by harvesting intensity [44,78]. In Asia, the most commonly harvested species is *D. sugokai* Izuka, 1907 [80,81]. 

*Diopatra aciculata* is an important aquaculture species in eastern Australia and has been cultivated for more than 15 years for use in recreational fishing as bait and as food in the conditioning of prawn broodstock of *Penaeus* spp. [82]. Interestingly, *D. aciculata* is also harvested as a bait species in South Africa, occurring in the Knysna Estuary, where it has been known as the moonshine worm for the last three decades [83]. A review of the Knysna Estuary bait industry is presented in this SI (see paper by Schoeman and Simon, 2023) [84].

## 8. *Diopatra* spp. as Alien or Introduced Species

Several *Diopatra* spp. have been considered alien or introduced species in Europe, South America, and the eastern Mediterranean Basin. In the Bay of Biscay—SW Europe—the distribution of *D. biscayensis* has been studied in depth, showing that it consists of four disjoint populations with the largest gap, consisting of 450 km, between the French populations of Vilaine/Loire and the Normano–Breton Gulf [85]. These authors concluded that the establishment of the Normano–Breton Gulf population could not have resulted from larval dispersal over this distance and considered human-assisted dispersal a likely explanation (Wethey et al., 2016) [56]. In the same way, the presence of *D. marocensis* along the coast of Turkey (from the Levantine and Aegean Seas) and the recently reported record of *D. neapolitana* from Brazil have been considered directly or indirectly human-mediated introductions [86,87]. However, the specific mechanisms, i.e., introduction vectors and pathways, are still poorly understood and far from being solved. 

An interesting case is *D. aciculata*. It was described in Port Phillip Bay, Melbourne, Australia by Knox and Cameron in 1971 [77]. It has been repeatedly stated that it is very similar morphologically to *D. neapolitana* [17,83,88]. A genetic analysis comparing *D. neapolitana*, *D. aciculate*, and *D. marocensis* indicated that *D. neapolitana* and *D. aciculata* are very close, with a 5% and 1% divergence for COI and 16S respectively [18]. The existence of baitworms resembling *D. neapolitana*/*D. aciculata* from the Knysna Estuary, Stellenbosch, South Africa [83] prompted a state-of-the-art morphological/genetic study [88] to ascertain its identity. However, the study could neither confirm nor disprove complete speciation and concluded that both species seem to be in the grey zone of speciation. Further studies are in the pipeline to discover the provenance of *D. aciculata*. Is it Australia or South Africa?

## 9. Regeneration and *Diopatra* spp. as Bioindicator Species

**Figure 7:**
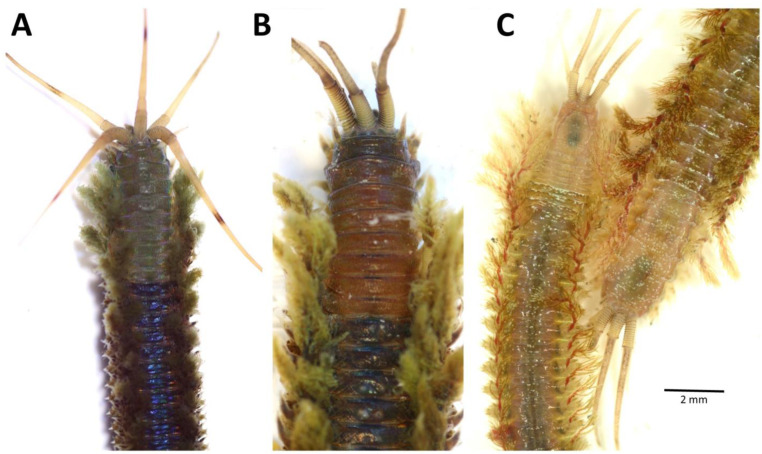
*Diopatra* spp. anteriorly regenerated specimens: (**A**) *D. dentata* (Sydney, SE Australia); (**B**) *D. neapolitana* (N Spain, Bay of Biscay); (**C**) *D. marocensis* (N Spain, Bay of Biscay).

Most polychaetes can regenerate lost appendages and the posterior end of the body, while some *Diopatra* spp. have the ability to regenerate even anterior segments and prostomial structures (Figure 7), as has been shown in *D. sugokai* (as *D. amboinensis*) by Pflugfelder (1929) [26]. Pires et al. (2012) [89] studied whether *D. neapolitana* (Figure 6B) can regenerate body damage caused by bait digging or predation and subsequently found that the species’ regenerative capacity proved to be affected by abiotic factors, such as seawater pH, temperature, or salinity, and thus can be used as sensitive markers to assess the metabolic effects of current climate change on marine invertebrate species [90]. In connection with studying bait collection in the Knysna Estuary, South Africa, the in situ incidence of regeneration in *D. aciculata* was investigated [84]. Although the species has a great capacity for regeneration, the small percentage of recovering worms does not negate the effects of bait collection (see paper by Schoeman and Simon, 2023 [84] in this SI). 

Some species, such as *D. neapolitana*, are excellent bioindicators of metal contamination, organic matter enrichment, and drugs (e.g., several pharmaceuticals such as paracetamol) in marine environments [9,91]. The interplay of seasonality, major and trace elements, and their impacts on *D. neapolitana* is presented in this SI (see paper by Giménez et al., 2022) [92].

## 10. *Diopatra* as a Source of Bioactive Compounds with Therapeutic Potential

Although *Diopatra* spp. have been studied for applied purposes for nearly two decades, they have not been explored for biotechnological/therapeutic purposes until very recently. In 2018, Jin Kim et al. [93] demonstrated the fibrinolytic and anticoagulation properties of a novel serine protease extracted from *D. sugokai*. This protease has a strong indirect thrombolytic activity over wide pH and temperature ranges and does not produce cytotoxicity in endothelial cells. Thus, this enzyme has a potential use in human thrombolytic therapy against ischemic stroke or brain ischemia and is worthy of further research as an alternative to current treatments for this cerebrovascular disease. Another Asian species of *Diopatra*, *D. claparedii*, was studied for the detection and assessment of bioactive compounds. This species has a great ability to regenerate both anterior and posterior parts upon self-amputation or injury, suggesting a wound-healing potential that was confirmed via the analysis of its aqueous extract, revealing that some metabolites are responsible for its wound-healing properties on acute wound model in rats [94]. Furthermore, the aqueous extract demonstrated antibacterial activities against *Escherichia coli* and *Pseudomonas aeruginosa*, thus making the *D. claparedii* extract a potential alternative as a natural healing promoter [94].

Likewise, *D. claparedii* can be utilized as a reducing agent in the biosynthesis of gold nanoparticles (AuNPs) [3]. The AuNPs possess outstanding physiochemical properties and are employed in a variety of applications in biomedical and pharmaceutical activities. *Diopatra claparedii* biosynthesized AuNPs (DioAuNPs) have antibacterial effects on several species of *Staphylococcus*, *E. coli* and *Salmonella typhi* [95].

The therapeutic and anti-infective (antibacterial) potential presented by the aforementioned *Diopatra* extracted compounds has opened up a vast field of interest for researchers and companies attracted to prospecting for valuable bioactive compounds produced by *Diopatra* species.

## 11. Climate Change, Geographic Distribution Changes, Dispersal

The European littoral zone, because of its rich historical record of species distribution, was studied to show the effects of climate change on the distribution of *Diopatra* spp. [56,96,97]. These authors conducted geographical surveys of *Diopatra* from 2006 to 2014 and compared them with historical records, concluding that the northern geographic limit of *Diopatra* spp. had advanced 300 km in France since 1893. Wethey et al., 2016 [56] explored the question of whether the disjunct distributions of *D. biscayensis*, in particular, could have been the result of historical refugia during cold periods and whether climate offered an explanation for its southern limit in northern Spain. They found no support for either of these hypotheses. Models of this type allow for exploration of biogeography using historical records and climate reconstructions and forecasting. In the case of the entire genus, much of this exploration is limited by the paucity of information available on physiological limitations. Much of what is known is only known for *D. cuprea*: failure of tube-building and maintenance at temperatures below 1.8 °C [98], cessation of feeding responses at ≤5 °C [57], and death at temperatures above 37.4 °C to 42.5 °C depending on latitude [99]. For *D. biscayensis*, the correlation of northern limits with summer seawater temperatures below 18 °C suggested a reproductive limitation [97], but no independent data exists. As far as we know, comparable information exists for none of the other species of *Diopatra*. Hopefully, this special issue will stimulate more answers as to limitations.

The genus serves a critical role as an ecosystem engineer, yet is under continued pressure from bait harvesting in all locales investigated (e.g., Italy [100]; Japan [81]; Portugal [78,89]; South Africa [101]; and Turkey [44]) and often is transported across national borders, e.g., [79,100,102]. Local populations in Portugal disappeared for several years due to harvest pressure ([89], pers. obs. Woodin); densities in Izmir Bay in Turkey showed dramatic reductions [44]. In several cases, it appears that the geographic distribution of species of *Diopatra* has been affected by human-assisted transport (e.g., *D. biscayensis* in France [56,85]; *D. neapolitana* in Brazil [87]; *D. aciculata* in South Africa and Australia [88]).

An important aspect of distribution and recovery from population decline is dispersal. Given the impact of climate change and the recognition of community structure changes due to invasive species, a focus of research attention is the dispersal potential of species. The probability of dispersal and the ability of larvae or some other dispersal stage of a species to cross distances on the order of 50 km or more in one reproductive season has long been recognized as a critical determinant of recovery from catastrophic events such as the winter of 1963 [103,104], as well as habitat or range expansion opportunities due to changes in physical conditions that increase the suitability of a locale (Southward 1967, 1991) [105,106]. The barnacle *Semibalanus balanoides*, for example, can expand its range over 100 km given changes in winter conditions (Wethey et al., 2011) [96]. No members of the genus *Diopatra* are known to be able to rapidly exploit changing conditions, as larval dispersal distances are very limited (Table 1). In all three of the large North Atlantic species, *D. cuprea*, *D. neapolitana*, and *D. biscayensis*, none has a larval period longer than several days (Table 1); therefore, dispersal is likely <10 km [85]. Out of the 67 described species, the larval types of ~14 species are known; seven brood their young within the tube and of those, five are known to have crawl-away juveniles with 15 chaetigers or more (*D. lilliputiana* Paxton, 1993 [17], *D. marocensis* Paxton et al., 1995 [38], *D. nishii* Paxton, 2014 [107], *D. tuberculantennata*, and *D. variabilis* Southern, 1921 [108]). One of the 14, *D. biscayensis*, apparently produces a mixture of 4- to 5-chaetiger larvae; some emerge from the egg mass and crawl away, often building a first tube on the tube of the adult, while others are positively phototactic and appear to have a short dispersal period (Arias and Paxton, 2015) [11]. *Diopatra albimandibulata* Paxton, 1993 [17] and *D. maculata* Paxton, 1993 [17] brood their young in a gelatinous egg sac/matrix attached to the parental tube and most likely have a short dispersal period after departing the egg sac. The other five species (*D. aciculata*, *D. cuprea*, *D. neapolitana*, *D. ornata*, and *D. sugokai*) are known to release positively phototactic larvae which spend three to six days in the water column, settling as 4- to 6- chaetiger larvae. No species thus are known with larvae likely to disperse long distances; however, several species have highly disjunct distributions, perhaps due to human-assisted transport (*D. aciculata*, *D. biscayensis*, *D. neapolitana*).

The new phylogeny for *Diopatra* in this volume [24] is a Bayesian analysis of both molecular and morphological data. Of the 14 species of which we know larval type (Table 1), eight are used in the phylogeny. Hektoen et al. (2022) [24] resolved five clades. The first is well supported and has an exclusive synapomorphy of ventral parapodial lobes; both *D. aciculata* and *D. neapolitana* are in clade 1, both with planktonic larvae. The other three species with planktonic larvae (*D. cuprea*, *D. ornata*, *D. sugokai*) plus *D. biscayensis* are in clade 5. The two species known to have crawl away brooded larvae that are in the phylogeny are in clades 2 (*D. marocensis*) and 4 (*D. tuberculantennata*). The implication is that these larval types represent a homoplasy, not a synapomorphy.

## 12. Conclusions

The genus *Diopatra* is known as an important ecosystem engineer in sedimentary systems, creating refugia and stabilizing sediments when in sufficient density [3,7,64,74,75]. It is also an important prey item for a variety of predators and used worldwide as bait [44,70,71,72,78,84]. However, our knowledge of the genus is quite limited as was revealed recently when the large and common onuphid of the Atlantic coast of Europe was found to be a protandric sequential hermaphrodite [12]. Efforts to predict the success or failure of *Diopatra* under future climatic scenarios or to ask questions about likely sources of species are severely hampered by our lack of basic physiological knowledge about almost all of the members of the genus with the exception of *D. cuprea.* This special volume dedicated to *Diopatra* will hopefully stimulate more investigations and further insights.

## Data Availability

Not applicable.
