# Correction: Arias et al. An Introduction to *Diopatra*, the Amazing Ecosystem Engineering Polychaete. *Biology* 2023, *12*, 1027

**DOI:** 10.3390/biology13010046

**Published:** 2024-01-15

**Authors:** Andrés Arias, Sarah A. Woodin, Hannelore Paxton

**Affiliations:** 1 Department of Organisms and Systems Biology (Zoology), University of Oviedo, 33071 Oviedo, Spain; 2 Department of Biological Sciences, University of South Carolina, Columbia, SC 29208, USA; woodin@biol.sc.edu; 3 School of Natural Sciences, Macquarie University, Sydney, NSW 2109, Australia; hannelore.paxton@mq.edu.au; 4 Australian Museum Research Institute, 1 William Street, Sydney, NSW 2010, Australia

The authors wish to make an erratum to the published version of the paper [1]. In the original article, the legend of Figure 5 was incompletely referenced. It should be as follows:

**Figure 5:** Larval development up to juvenile stage of *D. neapolitana* from the Bay of Biscay. Line drawings modified from Cazaux [49] and micrographs modified from Bergamo [50].

Ref. 50: Bergamo, G. Taxonomia, Reprodução e Desenvolvimento de *Diopatra neapolitana* Delle Chiaje, 1841 (Onuphidae, Annelida). Master Thesis, University of São Paulo, São Paulo, Brazil, 2018.

With this correction, a new reference is added and the order of some references has been adjusted accordingly.

The authors apologize for any inconvenience caused and state that the scientific conclusions are unaffected. This correction was approved by the Academic Editor. The original article has been updated.
